# Brucellosis knowledge, attitudes and practices of a South African communal cattle keeper group

**DOI:** 10.4102/ojvr.v86i1.1671

**Published:** 2019-02-18

**Authors:** Alicia Cloete, Cornelia Gerstenberg, Natalie Mayet, Stefano Tempia

**Affiliations:** 1 South African Field Epidemiology Training Programme, National Institute for Communicable Diseases, University of Pretoria, South Africa; 2 The School for Health Systems and Public Health, University of Pretoria, South Africa; 3 Department of Agriculture, Forestry and Fisheries, Animal Health, Pretoria, South Africa; 4 South Africa Regional Global Disease Detection Centre, National Institute for Communicable Diseases, South Africa; 5 Center for Respiratory Diseases and Meningitis, National Institute for Communicable Diseases, South Africa; 6 Influenza Division, Centers for Disease Control and Prevention, United States of America; 7 Influenza Program, Centers for Disease Control and Prevention, Pretoria, South Africa

## Abstract

Brucellosis remains an animal and public health concern in South Africa, given the intensity and widespread distribution of outbreaks in cattle. We conducted a cross-sectional survey among cattle keepers in the Whittlesea community of the Eastern Cape Province of South Africa, which utilises communal grazing. Individual cattle keepers (*N* = 227) who attended prearranged meetings in selected villages were interviewed using a structured questionnaire to assess their knowledge, attitude and practices (KAP) regarding bovine brucellosis. We compared KAP scores between previous brucellosis-affected villages and unaffected villages. We compared attitude and practices scores between those who had heard of brucellosis and those who had not and between those above the 75th percentile knowledge score and those below. The KAP for the study population were described using frequency tables. Scores of different groups were compared using the Welch *t*-test or the Wilcoxon rank-sum test. Knowledge scores of those who had heard of brucellosis (60%) showed a bimodal distribution with a 0/18 primary peak and 5–6/18 secondary peak. Attitude scores showed a median of 7/14 (interquartile range [IQR] 6–9), with 98% requesting more information on brucellosis. Practices scores showed a median of 6/18 (IQR 3–8), with high-risk practices identified that could facilitate brucellosis transmission. There were significant differences in attitude and practices scores between the groups above and below the 75th percentile knowledge score. The community showed poor knowledge, poor to average practices and average to good attitude. Identified high-risk practices highlight the risk of potential introduction and transmission of brucellosis between cattle and zoonotic transmission to humans.

## Introduction

Brucellosis has been identified as a neglected tropical disease by the World Health Organization (WHO 2006). Globally, it is regarded as the most common zoonotic infection among humans and remains endemic in many countries (Franco et al. 2007; Pappas et al. 2006). In resource-limited countries, communities and individuals who rely on livestock keeping for their livelihood are more at risk of zoonotic exposure because of close contact with livestock, and they may be less likely to be diagnosed and treated correctly (Marcotty et al. 2013; WHO 2006). Knowledge on brucellosis and the impact thereof is thought to be lacking among livestock farmers across all levels of production in South Africa (Department of Agriculture, Forestry & Fisheries [DAFF] 2017; National Department of Agriculture [NDA] 1997).

### Literature review

#### Brucellosis background

*Brucella abortus* mainly affects cattle, which clinically may present with abortion, reduced fertility, decreased milk production, orchitis and joint problems (Olsen & Tatum 2010; WHO 2006). *Brucella abortus* may be transmitted to humans mainly through the ingestion of non-pasteurised dairy products and through direct contact with birth material from infected cattle (Olsen & Tatum 2010; Zinsstag et al. 2007). Awareness of the disease, vaccination and testing of cattle, quarantine of infected herds and slaughter of positive reactors are crucial aspects of brucellosis control in cattle (Godfroid et al. 2004; Olsen & Tatum 2010). Zoonotic transmission should be mitigated mainly through disease control in livestock (Olsen & Tatum 2010) and also through the pasteurisation of milk (WHO 2006; Zinsstag et al. 2007).

#### Brucellosis in South Africa

A recent publication by the South African DAFF stated that there is currently great concern for brucellosis transmission given the intensity and widespread distribution of outbreaks in cattle throughout the country (DAFF 2017). Established brucellosis control measures in South Africa are currently undergoing discussion and revision to optimise collaboration between government, industry and farming communities (DAFF 2017). South African legislation states that all heifers between the ages of 4 and 8 months should be vaccinated with an efficient remedy (National Department of Agricultural Economics and Marketing 1986), of which the *B. abortus* Strain 19 and RB51 vaccines are currently registered (DAFF 2016). Livestock farmers are responsible for ensuring that their cattle herds are vaccinated and government veterinary services assist farmers where resources allow (DAFF 2017). At the moment, most cattle farmers are at risk of acquiring brucellosis-positive cattle in their herds, as there is generally poor compliance with brucellosis vaccination requirements, and testing is not currently compulsory for all cattle (DAFF 2017). Furthermore, a lack of proper fencing, mixing of animal groups and communal grazing practices create potential risks of spread among livestock in areas where communal grazing is practised (Alusi 2014; NDA 1997). Regarding zoonotic transmission, human cases of brucellosis are considered under-diagnosed and under-reported in the country, as is the case in many resourced-limited countries where brucellosis is endemic in cattle (Wojno et al. 2016).

#### Brucellosis knowledge, attitude and practices

Several brucellosis knowledge, attitude and practices (KAP) studies have been conducted and published in other resource-limited countries including Tajikistan, Jordan, Egypt, Uganda, Nigeria and Kenya. Knowledge, attitude and practices surveys provide critical and relevant information that help to explore potential risk factors, as well as potential intervention and prevention strategies for disease. Studies conducted in Uganda (Kansiime et al. 2014; Nabirye et al. 2017), Kenya (Obonyo & Gufu 2015), Jordan (Musallam, Abo-Shehada & Guitian 2015), Nigeria (Buhari et al. 2015) and Tajikistan (Lindahl et al. 2015) highlighted the need for brucellosis education for improved prevention, management and control of the disease. Each study showed particular KAP trends and highlighted the importance of such studies to understand country-specific circumstances in order to address specific shortcomings.

#### Statement of the problem

There is currently a paucity of information on brucellosis in humans and livestock in South Africa, as well as on the brucellosis KAP in different farming systems, including communal grazing settings. The current risk of community members acquiring the disease from communal livestock is unknown and may only be extrapolated from the information available from other countries. A better understanding of the KAP regarding brucellosis could increase the understanding of brucellosis risk factors for cattle and humans, influence local awareness programmes and guide policy on brucellosis control interventions (Lindahl et al. 2015).

#### Objectives

We aimed to determine the current KAP of cattle keepers regarding bovine brucellosis, in the Whittlesea community of the Eastern Cape Province of South Africa where communal grazing of cattle is practiced. We also sought to compare the KAP of cattle keepers between previously brucellosis-affected and unaffected villages; to establish if there was any significant difference between attitude and practices scores between those who had heard and not heard of brucellosis previously, as well as between those who scored above the 75th percentile knowledge score and those who scored below.

#### Contribution to field

This study was conducted to gather critical information that is important for the understanding of potential zoonotic transmission of bovine brucellosis in communal farming settings in South Africa. The information obtained may be utilised to guide local awareness programmes and policy on disease control interventions to promote both cattle and human health.

## Research method and design

### Setting

**FIGURE 1:**
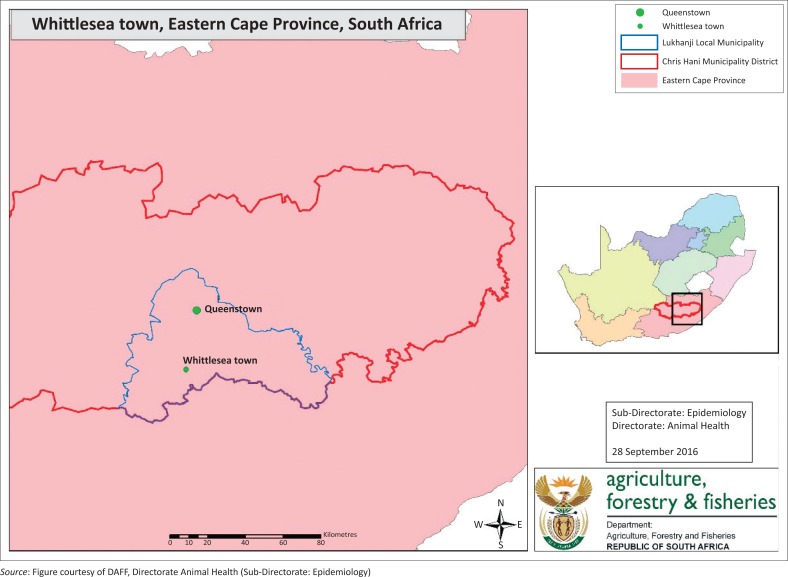
Location of the Lukhanji municipal area of the Chris Hani district in the Eastern Cape Province of South Africa.

The Whittlesea community consists of 48 villages that are located in the Lukhanji Local Municipality of the Chris Hani District in the Eastern Cape Province of South Africa (Figure 1), with an estimated human population of 56 500 in 2011 (Statistics South Africa). Communal land lies in between the scattered villages where sheep, goats and cattle from adjacent villages are grazed together. The Whittlesea community is serviced by the local Queenstown State Veterinary Office, which is part of the Eastern Cape Province Government Veterinary Services. During 2008–2009, Eastern Cape Province Government Veterinary Services succeeded in eradicating *B. abortus* from a communal cattle population after an outbreak occurred in the Lukhanji municipal area (Fisher 2010). Nine out of the forty-eight villages of the Whittlesea community had laboratory-confirmed brucellosis-positive cattle in the 2008–2009 outbreak, and extensive awareness campaigns were conducted throughout the area (Fisher 2010). Because of an intense and timeous disease control response, further spillover to cattle that originated from other villages was prevented. Brucellosis eradication from the affected villages was achieved through a combination of testing, slaughtering, vaccination, retesting and movement control measures.

### Design

We conducted a cross-sectional survey using a structured questionnaire to investigate KAP regarding brucellosis and associated risk factors among cattle keepers (representing their households) in the Whittlesea community. This community was selected for the study given the communal grazing setup, its history of bovine brucellosis infection and the existing regular interaction between livestock keepers and local government veterinary services.

### Study population and sampling strategy

The cattle keeper population of the Whittlesea community was extrapolated from official vaccination records. In 2016, the Queenstown State Veterinary Office vaccinated 20 419 cattle against anthrax, and we estimated an average of 10 cattle per owner, leading to a calculated population size estimate of 2042 cattle keepers. These cattle and cattle keepers originated from the 48 villages of Whittlesea community but were considered as a single epidemiological unit because of free movement and intervillage contact. The required study sample size was calculated as 323 cattle keepers, assuming a 50% proportion of any investigated KAP attributes at 95% confidence intervals and 5% precision, using Epi Info™ 7 (Centers for Disease Control and Prevention, GA, United States of America [US]).

The Whittlesea community was approached as a unit through a meeting with the village and community leaders. Through Queenstown State Veterinary Office we established that the most effective and efficient means of sampling would be to conduct cattle keeper meetings, using the villages as conjugation points. We selected a total of 18 of the 48 villages (37.5%) of the Whittlesea community: all villages that had laboratory-confirmed brucellosis-positive cattle during the 2008–2009 outbreak were purposefully selected (nine in total), and nine previously negative villages were selected through simple random sampling without replacement, using Microsoft^®^ Excel 2010 (Microsoft Corporation, Redmond, WA, US). The presumed negative villages tested negative for brucellosis during the 2008–2009 outbreak and during subsequent years. The meeting venues were selected for convenience to enable maximum contact with cattle keepers from the Whittlesea community with the available resources. For each of the 18 participating villages, all eligible cattle keepers who attended prearranged cattle keeper meetings were interviewed. An estimation of a good attendance of approximately 20 cattle keepers per meeting was envisioned to meet the calculated sample size.

### Procedure

A pretested KAP survey questionnaire consisting of 49 questions (some with additional subquestions) was used as the data collection tool. It was divided into five sections: (1) demographics; (2) knowledge on brucellosis as a disease; (3) attitudes towards animal health, human health and brucellosis; (4) self-reported practices relating to meat and dairy product consumption; and (5) self-reported practices relating to cattle husbandry. The questionnaire contained binary, multiple selection and open-ended questions. We adapted the questionnaire from a brucellosis KAP survey conducted in Tajikistan (Lindahl et al. 2015) to fit a rural South African context. We also added questions to capture necessary information on human health-seeking behaviour, communal grazing practices and information transfer routes. (The full questionnaire is available from the author or publisher).

The team of interviewers consisted of five animal health technicians (AHTs) from the Queenstown State Veterinary Office who were trained by the principal investigator to successfully interview participants using the questionnaire. The AHTs also pretested the draft KAP survey questionnaire on five random cattle keepers during the initial site visit in 2016 before the questionnaire was further adapted and finalised for use. The questionnaires were translated verbally into Xhosa by the interviewers as required. Two teams consisting of two to three interviewers conducted two meetings per day to cover the 18 villages during a specified 5-day study period in 2017.

Cattle keepers originating from the selected villages were interviewed. All interviewees were aged 18 years and older and able to communicate verbally in either English or Xhosa. A cattle keeper was referred to as a person who owns cattle or a person responsible for the cattle on the owner’s behalf. Cattle keeper meetings were organised in the selected villages on prearranged dates and times. Participants were consecutively enrolled in the study and interviewed as they arrived at the central meeting point. Afterwards an informative talk on brucellosis was conducted with a question-and-answer session.

### Analyses

Questionnaire responses were captured and coded using STATA version 14 (StataCorp, College Station, TX, USA). Missing data were excluded for the KAP analyses. A denominator was thus defined for each individual question to illustrate the total number of responses received and recorded for the KAP questionnaires. Descriptive statistics were used for demographic characteristics and brucellosis KAP of the respondents.

We allocated scores to specific questions in the KAP survey questionnaire following a scoring sheet developed by brucellosis experts of DAFF. (The scoring sheet is available from the author or publisher.) For the knowledge section, we distinguished between participants who stated they had heard of brucellosis previously and those who had not. Only the participants who indicated they had heard of brucellosis were further assessed in the knowledge section, and a maximum score of 18 could be achieved from 9 questions. A participant could achieve a maximum score of 14 from 9 questions in the attitude section and a maximum score of 18 from 11 questions in the practices section. A higher score would imply better attitude and practices, respectively. No previous benchmark scores were available for comparison. In addition, we categorised the respondents’ level of knowledge, attitude and practices, respectively, as high or moderate to low using the 75th percentile of the individual scores for further analysis.

The Welch *t*-test or the Wilcoxon rank-sum test was used to assess differences between knowledge, attitude and practices scores, respectively, between brucellosis-affected and unaffected village groups. The Welch *t*-test or the Wilcoxon rank-sum test was also used to assess differences between attitude and practices scores between those who had heard and not heard of brucellosis previously, as well as between those who scored above and those below the 75th percentile knowledge score. STATA version 14 was used to perform statistical analyses.

### Potential benefits and hazards

No harm was associated with collecting information on KAP regarding brucellosis and associated risk factors. The potential gains included using the opportunity to also educate the community regarding brucellosis during the contact sessions.

### Recruitment procedures

Participants were recruited on a voluntary basis as they arrived to attend prearranged cattle keeper meetings.

### Informed consent

An information sheet and informed consent document was used by the interviewers to explain the study process and purpose and to obtain consent. The study was voluntary and anonymous.

### Data protection

Questionnaires were administered once off, and the participants’ names and contact details were not collected. A unique identifier was assigned to each participant and used as a study number to aid data collection and capturing. The completed questionnaires and the consent forms have been stored securely by the principal investigator. The electronic database has been securely stored by the principal investigator, and only co-authors have access to the database.

## Results

### Socio-demographic characteristics of respondents

A total of 227 cattle keepers were interviewed during the study period, 73% of whom were male (Table 1). The median age of cattle keepers was 62 years (interquartile range [IQR] 51–71 and range of 21–93). The median number of household members was 4 (IQR 3–5 and range of 1–11). In terms of household education level, 41.6% (94/226) of respondents indicated some level of primary school, 46.0% (104/226) some level of secondary school and 12.0% had completed tertiary education. The predominant language in the households was Xhosa (99.1%). The median travel distance to a healthcare facility was 1 km (IQR 1–4 and range of 0–40). The median number of cattle kept by each household was 10 (IQR 5–19 and range of 1–313). Many of the cattle keeper households also owned goats (63%) and/or sheep (55%).

**TABLE 1:** Selected socio-demographic characteristics of cattle keeper households in the Whittlesea community, South Africa, 2017.

Variable (*N* specified because of missing data)	Frequency	
	*N*	%
**Gender of participant (*N* = 227)**		
Male	165	72.7
Female	62	27.3
**Age group of participant (*N* = 227)**		
18–30	11	4.9
31–50	43	18.9
51–70	116	51.1
> 70	57	25.1
**Highest education level in household (*N* = 226)**		
None	1	0.4
Primary level	94	41.6
Secondary level	104	46.0
Tertiary level (diploma and/or degree)	27	12.0
**Household size (*N* = 227)**		
> 3	47	20.7
3–6	150	66.1
> 6	30	13.2
**Number of cattle owned by household (*N* = 227)**		
< 5	49	21.6
5–20	128	56.4
> 20	50	22.0

### Knowledge on brucellosis as a disease in cattle and in humans

Out of 227 participants, 136 (59.9%) indicated they had heard of brucellosis previously. Table 2 shows the responses to some of the knowledge questions of these 136 participants. The main sources of information on brucellosis were veterinary services (53.7%; 73/136), community gatherings or talks (17.6%; 24/136), friends or family members (13.2%; 18/136) and radio or television (11.0%; 15/136). The majority of respondents reported having heard of brucellosis a year ago, with a noticeable peak detected for a response of 8–9 years ago as well.

**TABLE 2:** Knowledge of brucellosis among participants that reported having heard about the disease, Whittlesea community, South Africa, 2017 (reflecting the outcome of some of the knowledge questions).

Variable	Frequency	
	*N*	%
**Where did the participant hear of brucellosis† (*N* = 136)**		
Veterinary services	73	53.7
Community gathering and/or talk	24	17.6
Neighbours and/or friends and/or family	18	13.2
Radio and/or television	15	11.0
**Animals infected with brucellosis† (*N* = 130)**		
Don’t know	65	50.0
Cattle	48	36.9
Goats and/or sheep	31	23.8
All animals	23	17.7
**Transmission of brucellosis in cattle† (*N* = 128)**		
Don’t know	88	68.8
Abortion	25	19.5
Placenta from live births	23	18.0
Shared grazing	19	14.8
**Symptoms in cattle† (*N* = 125)**		
Don’t know	98	78.4
Abortion	24	19.2
Weak calves	15	12.0
Bull infertility	6	4.8
**Transmission of brucellosis in humans†,‡ (*N* = 18)**		
Drinking raw milk	12	66.7
Assisting with calving and/or handling placenta	4	22.2
Slaughtering an infected animal	3	16.7
Handling an abortion	2	11.1
**Symptoms in humans† (*N* = 118)**		
Don’t know	103	87.3
Fever	10	8.5
Headache	8	6.8
Flu-like symptoms	4	3.4

Note: *N* specified because of missing data.

† , Multiple answers allowed.

‡ , This question was asked only if participants indicated that ‘yes, humans can get infected with brucellosis’.

When asked which animals could become infected with *B. abortus*, 50.0% (65/130) of respondents did not know, 36.9% (48/130) stated cattle, 23.8% (31/130) sheep and goats and 17.7% (23/130) all animals. When asked about the transmission of brucellosis in cattle, 68.8% (88/128) of respondents did not know and 19.5% (25/128) stated abortion. Participants reported the following as signs of brucellosis in cattle: 19.2% (24/125) abortion and 12% (15/125) weak calves, whereas 78.4% (98/125) of respondents did not know. Only 16.8% (22/131) of respondents knew that humans could become infected with brucellosis. Among these respondents, drinking raw milk (66.7%; 12/18), assisting with calving or handling placenta (22.2%; 4/18), slaughtering an infected animal (16.7%; 3/18) and handling an abortion (11.1%; 2/18) were reported as possible means of zoonotic transmission. When asked about the symptoms of brucellosis in humans, 87.3% (103/118) of participants did not know, and 8.5% (10/118) cited fever.

The fact that brucellosis is a government-controlled disease was acknowledged by 55.6% (74/133) of respondents, and 50.0% (67/134) of respondents were aware of a brucellosis vaccine for cattle. The chronic nature of brucellosis in cattle was familiar to 30.1% (40/133) of respondents, whilst 9.0% (12/134) of respondents were aware of the availability of treatment for brucellosis in humans.

### Attitudes towards animal health, human health and brucellosis

When asked if they ensured that new cattle were healthy before buying or receiving them, 33.7% (71/211) responded positively (Table 3). To ensure cattle health, 37.5% (30/80) of respondents stated that they sought veterinary advice, 28.8% (23/80) bought from people they knew or trusted and 22.5% (18/80) relied on their own experience. When asked what action would be taken if a cow aborted, 46.2% (102/221) of respondents indicated that they would contact veterinary services for help, 21.7% (48/221) did not know what to do and 19.0% (42/221) would do nothing. Abortion in cattle was considered a serious condition by 57.8% (130/225) of respondents. When asked if they would get rid of cattle that had a chronic disease, only 20.4% (46/225) of respondents concurred.

**TABLE 3:** Responses of participants to attitude questions towards animal health, human health and brucellosis, Whittlesea community, South Africa, 2017 (reflecting the outcome of some of the attitude questions).

Variable	Frequency	
	*N*	%
**How health is ensured when receiving new cattle† (*N* = 80)**		
Seek veterinary advice	30	37.5
Buy from people known and/or trusted	23	28.8
Rely on own experience	18	22.5
**Action taken if a cow should abort† (*N* = 221)**		
Contact veterinary services for help	102	46.2
Don’t know	48	21.7
Nothing	42	19.0
Treat with home remedies	19	8.6
**Action taken if participant has flu-like symptoms† (*N* = 219)**		
Go to the doctor or clinic	204	93.2
Stay home and self-medicate	12	5.5
Don’t know	6	2.7
Go to the traditional healer	4	1.8
**Preferred information communication methods† (*N* = 216)**		
Farmers’ day meeting with veterinary services	126	58.3
Community meeting with veterinary services	118	54.6
Information pamphlet	9	4.2
Radio and/or television	7	3.2

Note: *N* specified because of missing data.

† , Multiple answers allowed.

The statement ‘raw milk is better and healthier than boiled (pasteurised) milk’ was agreed to by 46.9% (105/224) of respondents, whilst 62.4% (138/221) of respondents agreed to the statement ‘humans can get certain diseases from slaughtering animals’. When asked if participants considered it a serious situation if they could get sick from cattle that have brucellosis, 51.8% (115/222) of respondents did not concur. When asked if a participant would be willing to wear gloves if it helped to prevent disease transmission, 77.4% (123/159) concurred (this question was relocated from the practices section).

Participants were asked what action they would take if they experienced fever or influenza-like symptoms: 93.2% (204/219) of respondents replied that they would go to a health facility or doctor. The majority of respondents (98.7%; 220/223) stated that they would like to receive more information on brucellosis. The preferred reported methods of communication were as follows: farmers’ day meetings with veterinary services (58.3%; 126/216) and community meetings with veterinary services (54.6%; 118/216). These two options were commonly chosen as participants indicated that they were then able to ask questions as well. Only 4.2% (9/216) stated they preferred an information pamphlet, and only 3.2% (7/216) preferred information via radio or television.

### Practices relating to meat and dairy consumption and cattle husbandry

Milk was consumed by 89.0% (202/227) of respondents and 36.8% (74/201) of respondents stated that they consumed boiled or pasteurised milk exclusively (Table 4). Milk sources were indicated by respondents as follows: 80.0% (148/185) obtained milk from their own cows, 21.1% (39/185) from commercial stores and 20.5% (38/185) from informal stores. A majority of 84.8% (184/217) respondents indicated that it was practical to boil milk. Regarding meat practices, 83.1% (187/225) of respondents indicated that they practised home slaughter of cattle in their household.

**TABLE 4:** Responses of participants regarding practices relating to dairy consumption and cattle husbandry, Whittlesea community, South Africa, 2017 (reflecting the outcome of some of the questions on practices).

Variable	Frequency	
	*N*	%
**Conditions of milk consumed† (*N* = 201)**		
Boiled and/or pasteurised	107	53.2
Raw	79	39.3
Raw and soured	78	38.8
Exclusively boiled and/or pasteurised	74	36.8
**Milk sources† (*N* = 185)**		
Own cows	148	80.0
From commercial store	39	21.1
From informal store (spaza)	38	20.5
**Other dairy (cheese, yogurt, etc.) sources† (*N* = 187)**		
From commercial store	139	74.3
From family and/or friends	75	40.1
Homemade	6	3.2
**Caretaker of the cattle† (*N* = 220)**		
Owner	153	69.5
Shepherd and/or labourer	44	20.0
Other family member	39	17.7
**Cattle source^†^ (*N* = 222)**		
Bought from people in the community	109	49.1
Inherited and/or gift	59	26.6
Bought from other communities and/or areas	36	16.2
Bought from commercial farmers	16	7.2
**Who cattle are regularly sold to† (*N* = 152)**		
Local market and/or auctions	114	75.0
People in community	65	42.8
Abattoir	1	0.7
**Action taken if aborted foetus found† (*N* = 226)**		
Bury in ground	79	35.0
Give to dogs	58	25.7
Throw away or dump	24	10.6
Burn	18	8.0
Nothing	16	7.1
Report to AHT and/or state veterinarian	13	5.8
**Action taken if placental membranes found† (*N* = 220)**		
Bury in ground	81	36.8
Give to dogs	68	30.9
Throw away or dump	25	11.4
Burn	16	7.3
Nothing	11	5.0

Note: *N* specified because of missing data.

AHT, animal health technician.

† , Multiple answers allowed.

When asked about cattle origin, 49.1% (109/222) indicated that they had bought their cattle from people in the community, 26.6% (59/222) had obtained their cattle as an inheritance, 16.2% (36/222) had bought their cattle from other communities or areas and 7.2% (16/222) had bought their cattle from commercial farmers. Only 7.6% (17/225) of respondents indicated that they had purchased or received new cattle during the last year. In contrast, 60.9% (109/179) of respondents stated that they sold cattle on a regular basis. Of these, 75.0% (114/152) of respondents indicated that they sold to local markets or auctions, 42.8% (65/152) sold to people in the community, and only one participant indicated sale directly to an abattoir. Moreover, 98.2% (221/225) of respondents indicated that their cattle shared grazing and/or water with other livestock, and 91.5% (205/224) of respondents claimed that they could not keep their cattle separate from other livestock. Only 6.9% (15/218) of respondents indicated that they had inquired if cattle had been tested for brucellosis before they bought or received them.

Regarding assistance during parturition, 21.7% (49/226) stated that a household member had helped to deliver a calf and 22.9% (48/210) of respondents stated that a household member had handled placental membranes. In terms of action that would be taken if an aborted foetus was found, 35.0% (79/226) of respondents stated that they would bury the foetus, and 25.7% (58/226) would give it to dogs. Similarly, in terms of action that would be taken if placenta was found, 36.8% (81/220) of respondents stated that they would bury the placenta, and 30.9% (68/220) would give it to dogs. When asked if their cattle were vaccinated against brucellosis, 44.7% (101/226) of respondents stated yes, and 25.2% (57/226) stated that they did not know. When asked if their cattle had been tested for brucellosis before, 47.8% (107/224) of respondents stated yes, and 16.5% (37/224) stated that they did not know. From respondents who indicated their cattle had been tested, 86.8% (72/83) said that their cattle had tested negative, whilst 12.1% (10/83) did not know the testing results. The number of years ago that cattle were last said to be tested followed a bimodal pattern, with 49% (49/100) stating 1–2 years ago and 33% (33/100) stating 8 years ago.

### Knowledge, attitude and practices scores

**FIGURE 2:**
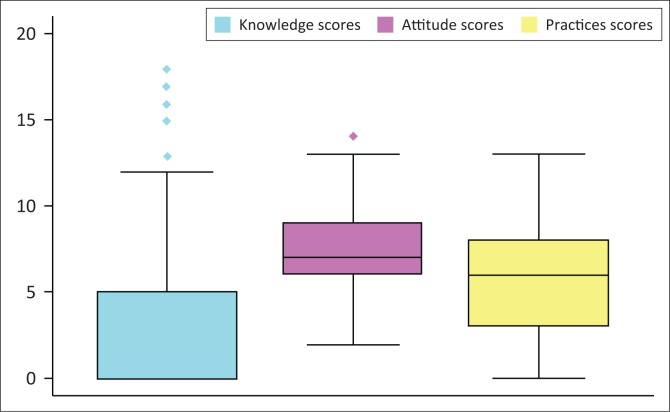
Boxplot showing knowledge, attitude and practices score distribution for communal cattle keepers of the Whittlesea community, South Africa, 2017.

For the knowledge section, 91 out of 227 (40%) participants indicated that they had not heard of brucellosis and were awarded a knowledge score of zero. The overall knowledge score of those who indicated that they had heard of brucellosis showed a bimodal distribution, with a main peak of zero and a second of 5–6 (out of a possible maximum of 18). For all participants, the overall median attitude score (out of a possible maximum of 14) was 7 (IQR 6–9), and the overall median practices score (out of a possible maximum of 18) was 6 (IQR 3–8). Knowledge, attitude and practices scores for all participants are illustrated in Figure 2, which shows comparative scoring between the three sections. Attitude scores were the highest, followed by practices scores and then knowledge scores.

### Comparison of scoring between different groups

No significant difference in the knowledge (Wilcoxon rank-sum test: *p*-value = 0.786), practices (Wilcoxon rank-sum test: *p*-value = 0.405) and attitude (Welch *t*-test: *p*-value = 0.553) scores was observed between previously brucellosis-positive and -negative villages.

There was no significant difference in the attitude (Welch *t*-test: *p*-value = 0.072) and practice (Wilcoxon rank-sum: *p*-value = 0.137) scores between respondents who had heard about brucellosis previously and those who had not.

There were statistically significant differences in the attitude (Welch *t*-test: *p*-value = 0.041) and practices (Wilcoxon rank-sum: *p*-value = 0.007) scores between respondents above and those below the 75th percentile of knowledge scores.

### Trustworthiness

#### Reliability

The structured KAP survey questionnaire was piloted prior to use during a previsit to the Whittlesea community. After the AHTs received training on questionnaire administration and data collection, a copy of the questionnaire was given to each to interview a random cattle keeper in the community. After completion of this exercise, the questionnaire was edited based on the findings and additional inputs provided by the AHTs. Prior to official interviews and data collection, the AHTs received repeat training by the principle investigator to promote uniformity of the interview and recording process.

#### Validity

All eligible cattle keepers who attended the prearranged meetings were interviewed. The overall turnout to the meetings was rated satisfactory by the AHTs who normally service the villages. Overall, only 227 cattle keepers were successfully interviewed, compared to the calculated sample size of 323. The smaller sample size may impact our power to detect ‘real’ differences.

## Discussion

Approximately 60% of respondents stated that they had heard of brucellosis previously. Our overall findings on the cattle keepers of the Whittlesea community showed that the knowledge levels were poor, resulting in poor to average practice scores regarding brucellosis, whilst the attitude score tended to be average to good. There were significant differences between the attitude (*p*-value = 0.041) and practices (*p*-value = 0.007) scores of the groups above and those below the 75th percentile knowledge score. This finding may suggest that poor practices were a result of poor knowledge rather than poor attitude in the study population.

The KAP scores did not differ significantly between previously brucellosis-affected and unaffected villages, confirming the initial assumption that there were no significance differences between the villages (sampling sites). Furthermore, the attitude and practices scores did not differ significantly between the groups who had heard of brucellosis previously and those who had not, suggesting that, to a certain extent, attitude and practices may be a result of community or cultural traits that are not influenced merely by individual awareness of brucellosis.

A relatively high number of participants stated they had heard of brucellosis previously (60%). Similar results were seen in brucellosis KAP studies conducted in northern Uganda (Nabirye et al. 2017) and Kenya (Obonyo & Gufu 2015), where 63% and 79% of community participants had heard of brucellosis, respectively. Brucellosis KAP studies conducted in Egypt (Holt et al. 2011), Nigeria (Buhari et al. 2015), Uganda (Kansiime et al. 2014) and Jordan (Musallam et al. 2015) showed that 83%, 93%, 99.3% and 100% had heard of brucellosis, respectively. Contrasting results were found in a brucellosis KAP study in Tajikistan, where only 15% had heard of brucellosis (Lindahl et al. 2015). The main source of brucellosis information was stated as unspecified media in the Jordan study (Musallam et al. 2015), community health workers in the Kenya study (Obonyo & Gufu 2015), parents in the Nigeria study (Buhari et al. 2015) and friends or family members in the Tajikistan study (Lindahl et al. 2015). Of particular interest in the Whittlesea community was that most had heard of brucellosis through veterinary services, indicating the importance of the role of government veterinary services in this regard. The bimodal distribution of when the participants had last heard of brucellosis probably reflected yearly interaction with government veterinary services on brucellosis and the dedicated awareness campaign during the last outbreak in 2008–2009.

The low overall knowledge scores of participants pointed toward a lack of detailed information and/or ineffective information transfer. Participants generally knew what government was doing regarding brucellosis, namely control and vaccination, but they lacked more in-depth knowledge with particular reference to zoonotic implications and disease prevention. Poor knowledge of brucellosis in humans specifically needs to be addressed in future awareness and education campaigns. Poor overall knowledge scoring was also reported in the Tajikistan (Lindahl et al. 2015), northern Uganda (Nabirye et al. 2017) and Nigeria (Buhari et al. 2015) brucellosis KAP studies, with poor to average knowledge reported by the Kenya study (Obonyo & Gufu 2015) and good knowledge scoring reported by the Jordan (Musallam et al. 2015) and Egypt studies (Holt et al. 2011). The Tajikistan (Lindahl et al. 2015), Kenya (Obonyo & Gufu 2015), Egypt (Holt et al. 2011) and Jordan (Musallam et al. 2015) studies showed good knowledge on brucellosis as a zoonosis, whilst the Nigeria study (Buhari et al. 2015) showed poor knowledge in this area.

We established that there were opportunities for improvement regarding attitude toward zoonotic disease transmission through slaughter practices and benefits of pasteurised or boiled milk consumption. Correct information on these practices is necessary to equip community members to safeguard themselves. Attitude toward the importance of abortion and especially chronic disease in cows also showed avenues for improvement, which would facilitate the identification of disease in these animals. The findings of human health-seeking behaviour patterns and access to human healthcare facilities showcased the importance of healthcare professionals, who should be equipped to enable identification and treatment of brucellosis in humans. Respondents showed a very positive attitude toward receiving more information on brucellosis, and it is critical for the relevant authorities to take note of preferred means of communication.

The overall attitude scores of participants were average to good in the Whittlesea community. Similarly, the brucellosis KAP study conducted in northern Uganda (Nabirye et al. 2017) showed a very positive attitude among community participants. In contrast, the Kenya study (Obonyo & Gufu 2015) found an unfavourable attitude among community participants. In the Tajikistan brucellosis KAP study (Lindahl et al. 2015), 63% of respondents requested more information on brucellosis, preferring an educational booklet; in contrast, the Kenya study (Obonyo & Gufu 2015) found that 97% requested more information and preferred the local FM radio stations for information transfer. These different information-channel preferences, as well as the different sources of current information, highlight the importance of establishing how a target community wants to be reached prior to conducting awareness or education campaigns.

Several high-risk practices were identified. Even though a greater sales trend was observed compared to new acquisitions of cattle, there was a lack of proper health investigation in purchased and received cattle, adding to the risk of brucellosis entry into the communal cattle population. This risk was further exacerbated by the overall disease transmission risk of the communal nature of grazing cattle. This highlights the importance of treating communal cattle as a single epidemiological unit and aiming disease prevention measures at keeping the communal herd protected against disease introductions. This would subsequently serve to prevent spillover to the human population. Similar high-risk practices of untested introductions and mixing with potentially infected cattle on communal grazing lands have been identified in Kenya (Obonyo & Gufu 2015), Nigeria (Buhari et al. 2015) and Uganda (Kansiime et al. 2014).

Potential zoonotic transmission risks were identified that could have led to brucellosis in humans if present in the cattle population. Raw milk was still being consumed, which could potentially be decreased through educating people on the benefits of boiling milk. Most households reported home slaughter of livestock. Slaughtering of brucellosis-infected cattle is considered a high-risk activity, as the persons conducting slaughter and handling contaminated meat may be exposed if precautions are not taken (Galinska & Zagórski 2013; Sadler 1960). Education on the proper handling and disposal of placenta and aborted foetuses is required to decrease potential human exposure to and environmental contamination with brucellosis. The positive attitude shown towards the use of gloves for protection should be noted by the relevant authorities and cattle keepers should take responsibility for using gloves as a measure of protection.

The practices results reflected a lack of effective communication during contact sessions between cattle keepers and government veterinary services in terms of what cattle were vaccinated and tested for. The ongoing brucellosis vaccination and testing of cattle creates an ideal opportunity to promote brucellosis awareness and to convey important information. The impact of the intense 2008–2009 brucellosis awareness and testing campaigns was reflected in the bimodal pattern of testing history, as stated by respondents, as well as the feedback on when respondents had last heard of brucellosis.

The overall practice scores of respondents were poor to average, with several high-risk behaviours identified in this community. Brucellosis KAP studies conducted in Tajikistan (Lindahl et al. 2015), Kenya (Obonyo & Gufu 2015), northern Uganda (Nabirye et al. 2017), Jordan (Musallam et al. 2015), Egypt (Holt et al. 2011) and Nigeria (Buhari et al. 2015) also revealed high-risk activities, including the handling of cattle birth material without protection. Tajikistan (Lindahl et al. 2015), Kenya (Obonyo & Gufu 2015) and northern Uganda (Nabirye et al. 2017) also reported the consumption of unpasteurised dairy products as a high-risk activity, whilst in the Jordan (Musallam et al. 2015) and Egypt (Holt et al. 2011) studies the majority of participants boiled milk before consumption but not always before making cheese.

### Limitations

Only 227 interviews out of the 323 (70%) calculated sample size were achieved. The lack of accurate recorded information on cattle and cattle keepers limited the validity of the sample size calculation. However, according to the local AHTs, the turnouts to the meetings were good compared to what is normally experienced. Cattle keepers who attended our meetings may have been more exposed to information compared to cattle keepers who do not routinely attend meetings. Recall bias may have occurred if participants had difficulties to recall information shared during previous information campaigns. Whereas the scoring system was developed and agreed upon by brucellosis experts of DAFF, the assigned questions’ scores may have been impacted by the perceived importance of KAP in the local context.

### Recommendations

The findings of this study contain useful information to understand the communal farming setting in the Whittlesea community that could be used to influence the approach to local brucellosis control and prevention strategies. Targeted, consistent brucellosis awareness and information campaigns could address the community’s high-risk practices by increasing their knowledge through utilising their positive attitudes and heeding the request for brucellosis information. The number of similarities and differences described between this study and other brucellosis KAP studies from different countries further highlights the importance of establishing KAP in local settings.

Existing contact sessions between government veterinary services and cattle keepers could be utilised optimally to create awareness of brucellosis and to provide relevant information on animal and human health and disease prevention. Information transfer would probably be more efficient if provided through methods that are acceptable to the community, which in this study was indicated as community or farmer’s meetings with government veterinary services. It would be of benefit if particular attention could be paid to improving knowledge of the community on brucellosis as a zoonotic disease and to ensuring that healthcare practitioners are equipped to identify and treat the disease. It is also useful to note that relative findings of this study could also be applicable to other zoonoses present in the country that have similar risk factors, for example *Mycobacterium bovis*.

Similar KAP studies could be used for the whole country to determine baseline KAP for different communities prior to embarking on awareness and education campaigns in order to adapt the approach and content thereof to optimise efficient and effective information transfer. Knowledge, attitude and practices studies could then subsequently be repeated as an evaluation tool to determine the effectiveness of conducted awareness and education campaigns.

## Conclusion

In conclusion, the cattle keepers of the Whittlesea community showed poor knowledge, poor to average practices and average to good attitude pertaining to brucellosis and related factors. Bovine brucellosis is currently prevalent throughout South Africa, leaving this community at risk of introducing brucellosis into the cattle population, as new cattle brought in from other locations are not always tested, and test results are generally not requested. If brucellosis-positive cattle enter the communal cattle population, there is a risk of spread between cattle as a result of communal grazing practices and a lack of fencing. This highlights the importance of treating communal cattle as a single epidemiological unit regarding disease prevention, detection and control. Community members are at risk of acquiring brucellosis if the disease is present in the cattle population through several high-risk practices, including consumption of raw milk and the handling of cattle birth material.

This KAP study provides necessary information to address shortcomings in knowledge and practices within the study area to improve both animal and human health.
